# Missed Opportunities for Early De-Escalation of Antipseudomonal Beta-Lactam Antimicrobial Therapy in Enterobacterales Bloodstream Infection

**DOI:** 10.3390/antibiotics13111031

**Published:** 2024-10-31

**Authors:** Mollie Reese, P. Brandon Bookstaver, Joseph Kohn, Casey Troficanto, Emily Yongue, Hana R. Winders, Majdi N. Al-Hasan

**Affiliations:** 1 Department of Medicine, University of North Carolina, Chapel Hill, NC 27599, USA; mollie.reese@unchealth.unc.edu; 2 Department of Clinical Pharmacy and Outcomes Sciences, University of South Carolina College of Pharmacy, Columbia, SC 29208, USA; bookstaver@cop.sc.edu; 3 Department of Pharmacy, Prisma Health Midlands, Columbia, SC 29203, USA; 4 Department of Medicine, University of South Carolina School of Medicine, Columbia, SC 29209, USA; 5 Department of Internal Medicine, Prisma Health Midlands, Columbia, SC 29203, USA

**Keywords:** bacteremia, antibiotics, gram-negative, sepsis, empirical therapy, outcome

## Abstract

**Background:** Antipseudomonal β-lactams (APBL) are commonly used for empirical therapy of Gram-negative bloodstream infections (BSI). This retrospective cohort study examines risk factors for prolonged APBL use (≥48 h) in patients with Enterobacterales BSI and compares 28-day mortality between early de-escalation of APBL and prolonged APBL therapy. **Methods:** Adult patients admitted to two community hospitals in South Carolina with Enterobacterales BSI from January 2010 to June 2015 were included in this study. Data were extracted manually from medical records. Multivariate logistic regression and Cox proportional hazards analyses were used to examine predictors of prolonged APBL therapy and mortality, respectively. **Results:** Among 993 patients with Enterobacterales BSI, 491 (49%) underwent early de-escalation of APBL and 502 (51%) received prolonged APBL therapy. Cancer, immune compromised status, residence at a skilled nursing facility, a high Pitt bacteremia score, non-urinary source of infection, and BSI due to AmpC-producing Enterobacterales were independently associated with prolonged use of APBL. Antimicrobial stewardship interventions were inversely associated with prolonged APBL use. Early de-escalation of APBL was not associated with increased mortality. **Conclusions:** This study exemplifies the safety and effectiveness of early de-escalation of APBL in Enterobacterales BSI. Antimicrobial stewardship strategies should be implemented to encourage the practice of early de-escalation of antimicrobial therapy, including in high-risk populations.

## 1. Introduction

Gram-negative bloodstream infections (BSI) are common worldwide, with the Enterobacterales family representing the overwhelming majority of these infections [1,2,3]. The overall case fatality rate of Gram-negative BSI ranges from 12% to 20% [2,4]. Antipseudomonal β-lactam (APBL) antibiotics are commonly used for empirical therapy of Gram-negative BSI, although *Pseudomonas aeruginosa* accounts for <10% of all Gram-negative bloodstream isolates in community hospitals [5]. Moreover, risk factors of *P. aeruginosa* BSI are well established and include nosocomial acquisition, severely immune-compromised hosts, and prior use of β-lactam antibiotics [5,6]. APBL therapy carries risks of high healthcare costs, toxicities, and the emergence of multidrug resistance (MDR) [7,8]. The rise of MDR Enterobacterales is a global threat, with counts increasing by nearly 10% worldwide from 1997 to 2013 [1,9,10]. Each additional day of APBL therapy increases the risk of selection of MDR bacteria, particularly MDR *P. aeruginosa* [8]. Infections with MDR bacteria are associated with increased mortality and prolonged hospitalizations raising concern about the use of APBL agents [9]. Additionally, the prolonged use of APBL is associated with an increased risk of *Clostridioides difficile* infections (CDI) [11,12]. The molecular and chemical aspects explaining the mechanism of induced toxicities and multidrug resistance associated with APBL have been described [13]. Although it is occasionally appropriate as a definitive therapy, APBL should often be de-escalated to a narrower therapy in the early days of treatment for Enterobacterales BSI to reduce these risks [14].

De-escalation of antimicrobial therapy from broad-spectrum to narrower spectrum agents has been a cornerstone of antimicrobial stewardship [7]. The question remains how early de-escalation is defined and outlined in clinical practice. Earlier studies evaluating the safety of early de-escalation defined it as the discontinuation of broad-spectrum antimicrobials within 96 h or after susceptibility results [15,16,17,18]. The availability of rapid diagnostics for the identification of bacteria and common antimicrobial resistance mechanisms in the bloodstream and other clinical specimens enabled early de-escalation of antimicrobial therapy to be redefined from 96 to 48 h [19]. Additionally, in an era when the total treatment duration for most serious infections has been reduced to 5–7 days [20], the use of 96 h of broad-spectrum agents now constitutes the majority of the total antimicrobial treatment course. In a landmark study, Bookstaver and colleagues demonstrated that the implementation of rapid diagnostics for BSI, namely, MALDI-TOF and multiplex PCR, coupled with antimicrobial stewardship live alerts and interventions reduced APBL treatment duration from 96 h to 48 h in patients with Enterobacterales BSI [19]. Subsequent work demonstrated that early de-escalation of APBL within 48 h in Enterobacterales BSI was associated with a three-fold decline in the risk of CDI [11]. The subsequent widespread use of rapid diagnostics for early identification of bloodstream isolates had a major impact on antimicrobial stewardship practices with further emphasis on the early de-escalation of antimicrobial therapy. However, the routine implementation of early de-escalation of APBL in Enterobacterales BSI may be challenging, particularly in certain patient populations where primary healthcare providers are accustomed to prescribing APBL and may feel uncomfortable using narrower-spectrum agents even when *P. aeruginosa* is not detected in blood cultures. Identification of these populations will inform antimicrobial stewards and help to develop specific interventions to enhance early de-escalation of APBL in these patients. Studies demonstrating the safety of early de-escalation of APBL, defined as within 48 h, have yet to be documented in the medical literature and it is equally important to foster confidence in this approach in clinical practice. The primary aim of this retrospective cohort study is to identify risk factors for prolonged APBL use (≥48 h) in patients with Enterobacterales BSI by comparing variables associated with prolonged APBL use to those associated with early de-escalation of APBL antibiotics. The secondary aim is to compare 28-day mortality in patients who underwent early de-escalation of APBL to those who received prolonged APBL therapy for ≥48 h.

## 2. Results

### 2.1. Demographic, Clinical, and Microbiological Characteristics

**Figure 1:**
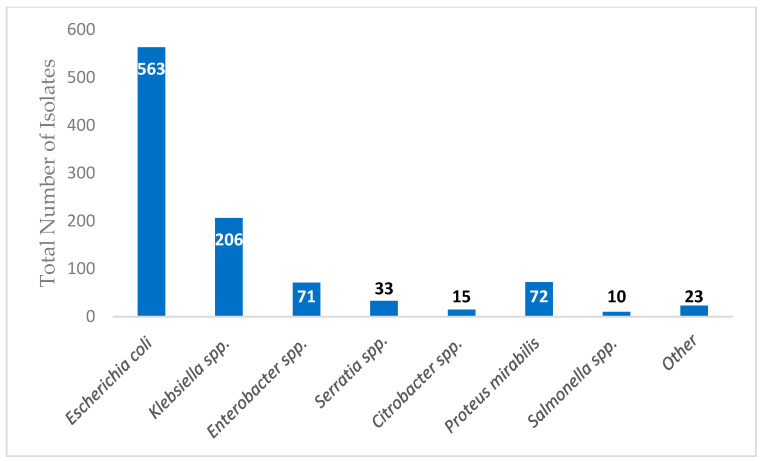
Microbiology of Enterobacterales bloodstream infection.

**Figure 2:**
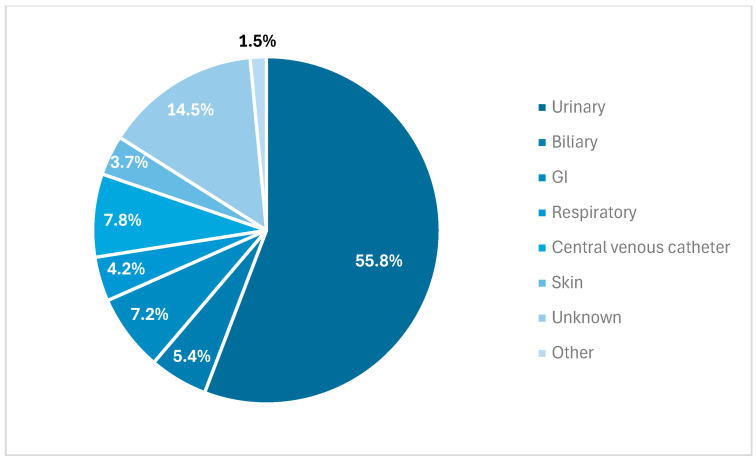
Source of Enterobacterales bloodstream infection. GI: gastrointestinal.

During the study period, 993 adult patients were hospitalized with the first episode of monomicrobial Enterobacterales BSI. Overall, the median age was 65 years and 539 (54.3%) were women. *Escherichia coli* (563; 50.6%) was the most common bloodstream isolate, followed by *Klebsiella* spp. (206; 20.7%), *Proteus mirabilis* (72; 7.3%), *Enterobacter* spp. (71; 7.2%), *Serratia* spp. (33; 3.3%), *Citrobacter* spp. (15; 1.5%), *Salmonella* spp. (10; 1.0%) and others (23; 2.3%), as demonstrated in Figure 1. The urinary tract was the most common source of BSI (554; 55.8%). Other sources of BSI are demonstrated in Figure 2.

Of this cohort, 491 (49.4%) patients underwent early de-escalation of APBL within 48 h and 502 (50.6%) received prolonged APBL therapy for ≥48 h. The baseline demographics and clinical characteristics of the two arms of this cohort are demonstrated in Table 1.

### 2.2. Risk Factors for Prolonged APBL Therapy

In the univariate logistic regression model, male sex, cancer, immune-compromised status, residence at a skilled nursing facility, hospital-onset BSI, non-urinary source of infection, BSI due to AmpC-producing Enterobacterales, and high Pitt bacteremia scores were associated with prolonged APBL therapy. ASP interventions were inversely associated with prolonged APBL use (Table 2). After adjustments in the multivariate model, male sex and hospital-onset BSI were not independently associated with prolonged APBL use. Cancer, immune-compromised status, residence at a skilled nursing facility, non-urinary infection source, BSI due to AmpC-producing Enterobacterales, and a high Pitt bacteremia score were independently associated with prolonged APBL therapy. ASP interventions remained independently associated with a shorter APBL duration (Table 3).

### 2.3. Predictors of Mortality

The crude 28-day all-cause mortality was 12.9% and 7.1% in patients who received ≥48 h and <48 h of APBL, respectively. The results of the univariate Cox proportional hazards regression for mortality are shown in Table 4. After adjustments for age, chronic comorbidities, acute severity of illness, and appropriateness of empirical antimicrobial therapy, early de-escalation of APBL was not associated with an increased risk of 28-day mortality (HR 0.91, 95% CI 0.57–1.46; *p* = 0.71) as demonstrated in Table 5.

## 3. Discussion

This cohort study emphasizes the safety and effectiveness of early de-escalation of APBL in hospitalized adults with Enterobacterales BSI and highlights antimicrobial stewardship opportunities for improvement in this practice. Early de-escalation from APBL to narrower-spectrum antibiotics occurred in approximately one-half (49.4%) of patients with Enterobacterales BSI in this study. Similar rates of de-escalation have been demonstrated in other cohort studies [17,18]. However, the clinical reasoning behind delaying de-escalation is poorly understood.

The study identified patients with cancer, immunocompromised status, residence at a skilled nursing facility, acute severity of illness (high Pitt bacteremia score), non-urinary source of BSI, and BSI due to AmpC-producing Enterobacterales as predictors of prolonged APBL use. Clinicians were reluctant to de-escalate APBL within 48 h in these patient populations despite a lack of detection of *P. aeruginosa* in the bloodstream by rapid diagnostics (multiplex PCR and/or MALDI-TOF). Cancer and immune-compromised status are well known risk factors for BSI due to *P. aeruginosa* [5,6]. Residents of skilled nursing facilities have frequent exposure to the healthcare system and antibiotics, which may increase the risk of *P. aeruginosa* colonization and infection [5]. High acute severity of illness and non-urinary sources of BSI imply lower margins of error due to the high predicted mortality in these patients [21]. It is possible that clinicians were concerned about the remote potential of late growth of *P. aeruginosa* in the bloodstream or other clinical cultures. The safety of early de-escalation of APBL demonstrated in this study will encourage clinicians to be more comfortable with early de-escalation of APBL in these populations in the future. As illustrated in Table 5, the current study demonstrated that mortality was comparable between patients who underwent early de-escalation of APBL and those who remained on APBL therapy for ≥48 h (HR 0.91, 95% CI 0.57–1.46; *p* = 0.71). This is consistent with the results of previous studies that also demonstrated the safety of the practice of de-escalation of antimicrobial therapy in Enterobacterales BSI, although these studies evaluated longer durations of empiric APBL therapy [17,22,23].

Early de-escalation of broad-spectrum antimicrobial therapy in persons living with cancer, other immune-compromising conditions, residents at skilled nursing facilities, and critically ill patients may have several advantages. These conditions are considered risk factors for CDI, which places these patients at higher risk of CDI compared to the general population [24]. Early de-escalation of APBL within 48 h has been associated with a three-fold decline in the risk of CDI in a previous study [11]. The potential decline in the risk of CDI has massive financial and public health advantages given the burden of CDI on the community and healthcare system [25]. Moreover, each additional day of APBL beyond 48 h has been associated with an increased risk of emerging antimicrobial resistance [8]. Early de-escalation of APBL may have major benefits in reducing the risk of developing antimicrobial resistance in these high-risk populations.

The study also demonstrated that antimicrobial stewardship interventions were successful in enhancing early de-escalation of APBL (OR 0.68, 95% CI 0.50–0.91; *p* = 0.02). This further supports the implementation of syndrome-specific antimicrobial stewardship interventions for BSI with live alerts and antimicrobial stewardship recommendations based on Gram stain and rapid diagnostic results. The implementation of syndrome-specific antimicrobial stewardship interventions has been associated with a decline in the use of broad-spectrum antimicrobial agents and hospital-onset CDI [26].

This study highlights the role of antimicrobial stewardship in the optimization of antimicrobial therapy. The local antimicrobial stewardship team recommended cefepime for empirical therapy of BSI due to *Enterobacter cloacae* and other potential chromosomal AmpC-producing Enterobacterales [14]. This explains the association between prolonged APBL use and BSI due to AmpC-producing Enterobacterales. This supports the antimicrobial stewardship principles of improving patients’ outcomes even when that entails the use of broad-spectrum antimicrobial agents in the right patient and the right setting.

The results of this study and the supporting literature emphasize that early de-escalation of APBL within 48 h is safe in the treatment of Enterobacterales BSI and urge healthcare providers to implement this concept in clinical practice. The development of evidence-based guidelines by multidisciplinary teams with the support of antimicrobial stewardship programs would encourage the practice of early de-escalation of antimicrobial therapy [27]. Education, the center of many antimicrobial stewardship efforts, emphasizing the safety and efficacy of early de-escalation of APBL in the treatment of Enterobacterales BSI will likely be necessary to improve adherence to this practice [27,28].

To our knowledge, this is the first study to examine the predictors of prolonged APBL therapy in Enterobacterales BSI. The large sample size of this cohort provided adequate power to examine both primary and secondary outcomes. The study has multiple limitations. Similar to other cohort studies, the current investigation may inherently be affected by unmeasured and unknown confounding variables. Antimicrobial resistance rates may have changed, and rapid diagnostics may have further advanced since the performance of this study. Additionally, data from one geographical region in the United States may not be generalizable to other parts of the world with vastly different antimicrobial resistance patterns and utilization of rapid diagnostic techniques.

## 4. Materials and Methods

### 4.1. Study Design and Objectives

The primary objective of this retrospective cohort study was to identify risk factors for prolonged APBL therapy (≥48 h) in hospitalized adults with Enterobacterales BSI. The secondary objective was to examine 28-day mortality in patients who underwent early de-escalation (within 48 h) versus those who received prolonged APBL therapy for ≥48 h. The study was conducted at the Prisma Health Richland and Baptist Hospitals in Columbia, South Carolina, USA. The two community-based teaching hospitals have a combined capacity of approximately 1000 beds. The study was approved by the Institutional Review Board at Prisma Health, and a waiver of informed consent was provided based on minimal risk.

### 4.2. Participants and Eligibility Criteria

This study included adults of 18 years of age or older who were hospitalized from 1 January 2010 to 30 June 2015 with Enterobacterales BSI (*n* = 1063). Patients less than 18 years of age, those with polymicrobial BSI, defined as >1 bacterial species in blood cultures, non-fermenting Gram-negative BSI, and those hospitalized for <48 h due to early death, discharge, or transfer were excluded from the study (*n* = 70).

### 4.3. Definitions and Data Collection

The clinical and laboratory standards institute guidelines and techniques were used to process the blood culture specimens in this study [29]. Appropriateness of antimicrobial therapy was determined by the susceptibility results from the Vitek^®^ 2 system (bioMérieux, Marcy-l’Étoile, France) [30]. The Centers for Disease Control and Prevention definitions were utilized to establish the source of BSI [31]. Piperacillin-tazobactam, ceftazidime, cefepime, imipenem-cilastatin, meropenem and aztreonam were the APBL (with or without beta-lactamase inhibitors) used during the study period. Early de-escalation of APBL was defined as a discontinuation of APBL within 48 h of the collection of the index blood culture and transitioning to a narrower spectrum antimicrobial agent. Prolonged APBL use was defined as a continuation of an APBL agent for ≥48 h from the onset of BSI. The Pitt bacteremia score was used to assess acute severity of illness [32]. Antimicrobial stewardship interventions were implemented on 1 January 2014 [19]. These interventions included the addition of rapid diagnostic testing, live alerts for positive blood cultures, recommendations for optimization of antimicrobial therapy, and systemwide Gram-negative BSI management guidelines [19].

Data were collected by manual review of the electronic medical records system and recorded in an Excel spreadsheet. Data points recorded in the study included age, sex, race, comorbidities, site of acquisition, medical comorbidities, acute severity of illness as measured by the Pitt bacteremia score, source of infection, microbiological results, antimicrobial therapy, and presence or absence of antimicrobial stewardship intervention. Internal validation of the data was performed through manual quality checks by a senior investigator (MNA). Redundant variables were collected to verify outcomes, such as severity of illness, as part of this internal validation.

### 4.4. Statistical Analysis

Multivariate logistic regression analysis was performed to evaluate the primary outcome of risk factors for prolonged APBL use. Variables associated with APBL use for ≥48 h in the univariate logistic regression analysis (*p* < 0.05) were included in the multivariate model using backward selection. Multivariate Cox proportional hazards regression was used to examine 28-day mortality. The multivariate Cox model was developed based on clinical relevance and statistical association with mortality in the univariate analysis (*p* < 0.05) using backward selection. Odds ratios (OR) and hazards ratios (HR) with 95% confidence intervals (CI) were used to describe the association of variables with the primary and secondary outcomes, respectively. Statistical significance was defined as a *p*-value < 0.05. JMP Pro version 17 (Carey, NC, USA) was used for statistical analysis.

## 5. Conclusions

This cohort study has identified major antimicrobial stewardship opportunities for the early de-escalation of APBL therapy, particularly in patients with cancer, other immune-compromising conditions, those who are critically ill, and those with high-inoculum infections. The current results are novel in demonstrating the safety and effectiveness of early de-escalation of APBL therapy in patients with Enterobacterales BSI, including high-risk populations. Clinical guidelines should be implemented in the management of Enterobacterales BSI to encourage clinicians to properly adopt early de-escalation of antimicrobial therapy. Additionally, antimicrobial stewardship interventions should be implemented to encourage the practice of early de-escalation of antimicrobial therapy in the general and high-risk populations.

## Figures and Tables

**Table 1:** Demographics and clinical characteristics of patients with Enterobacterales bloodstream infection by duration of antipseudomonal beta-lactam therapy.

Variable	APBL ≥ 48 h (*n* = 502)	APBL < 48 h (*n* = 491)
Age, median (IQR) [years]	65 (54–75)	66 (54–79)
Male sex, *n* (%)	260 (51.8)	194 (39.5)
Race, *n* (%)		
White	234 (46.6)	222 (45.2)
African American	247 (49.2)	254 (51.7)
Other	21 (4.2)	15 (3.1)
Diabetes mellitus, *n* (%)	200 (39.8)	194 (39.5)
End-stage renal disease, *n* (%)	55 (11.0)	44 (9.0)
Liver cirrhosis, *n* (%)	22 (4.4)	18 (3.7)
Cancer, *n* (%)	99 (19.7)	57 (11.6)
Immune compromised host, *n* (%)	65 (12.9)	33 (6.7)
Hospital-onset BSI, *n* (%)	133 (26.5)	84 (17.1)
Resident at SNF, *n* (%)	91 (18.1)	64 (13.0)
Urinary source of BSI	229 (45.6)	235 (66.2)
BSI due to AmpC-producing bacteria	92 (18.3)	50 (10.2)
Pitt bacteremia score, median (IQR)	2 (1–4)	1 (0–2)

APBL: antipseudomonal beta-lactams; IQR: interquartile range; BSI: bloodstream infection; SNF: skilled nursing facility.

**Table 2:** Risk factors of prolonged antipseudomonal beta-lactam therapy in the univariate analysis.

Risk Factor	Odds Ratio	(95% CI)	*p*-Value
Age (by decade)	0.97	(0.91–1.05)	0.46
Male sex	1.64	(1.28–2.12)	<0.001
White race	1.06	(0.82–1.36)	0.66
Diabetes mellitus	1.01	(0.79–1.31)	0.92
End-stage renal disease	1.25	(0.82–1.90)	0.29
Liver cirrhosis	1.20	(0.64–2.27)	0.57
Cancer	1.87	(1.31–2.66)	<0.001
Immune-compromised status	2.06	(1.33–3.20)	0.001
Residence at skilled nursing facility	1.48	(1.04–2.09)	0.03
Hospital-onset bloodstream infection	1.75	(1.28–2.37)	<0.001
Non-urinary source of infection	2.33	(1.81–3.02)	<0.001
Pitt bacteremia score (per point)	1.31	(1.23–1.41)	<0.001
Antimicrobial stewardship interventions	0.71	(0.54–0.94)	0.02

CI: confidence intervals.

**Table 3:** Predictors of prolonged antipseudomonal beta-lactam therapy in multivariate analysis.

Variable	Odds Ratio	95% (CI)	*p*-Value
Cancer	1.62	1.09–2.41	0.01
Immune compromised status	1.76	1.08–2.88	0.03
Residence at a skilled nursing facility	1.58	1.09–2.29	0.02
Non-urinary source of infection	2.10	1.58–2.79	<0.001
BSI due to Amp-C producing bacteria	1.56	1.04–2.35	0.02
Pitt bacteremia score (per point)	1.30	1.21–1.39	<0.001
Antimicrobial stewardship interventions	0.68	0.50–0.91	0.02

CI: confidence intervals.

**Table 4:** Risk factors of 28-day mortality in the univariate analysis.

Variable	Hazards Ratio	(95% CI)	*p*-Value
Age (by decade)	1.33	(1.16–1.53)	<0.001
Male sex	1.44	(0.95–2.19)	0.09
White race	2.13	(1.38–3.29)	<0.009
Diabetes mellitus	0.92	(0.60–1.41)	0.70
End-stage renal disease	0.85	(0.41–1.75)	0.65
Liver cirrhosis	2.05	(0.95–4.44)	0.07
Cancer	3.55	(2.31–5.45)	<0.001
Immune-compromised host	2.26	(1.33–3.83)	0.003
Residence at skilled nursing facility	1.49	(0.90–2.48)	0.12
Hospital-onset bloodstream infection	1.66	(1.06–2.59)	0.03
Nonurinary source of infection	1.75	(1.15–2.66)	0.009
BSI due to AmpC-producing bacteria	1.33	(0.77–2.28)	0.31
Pitt bacteremia score (per point)	1.32	(1.24–1.41)	<0.001
Inappropriate empirical antimicrobial therapy	2.18	(1.16–4.09)	0.016
Early de-escalation of APBL within 48 h	0.56	(0.36–0.86)	0.009

CI: confidence intervals; BSI: bloodstream infection; APBL: antipseudomonal beta-lactams.

**Table 5:** Predictors of 28-day mortality in the multivariate analysis.

Variable	Hazard Ratio	(95% CI)	*p*-Value
Age (by decade)	1.53	(1.30–1.80)	<0.001
Cancer	5.14	(3.27–8.10)	<0.001
Liver cirrhosis	3.20	(1.45–7.09)	0.004
Pitt bacteremia score (per point)	1.45	(1.34–1.56)	<0.001
Inappropriate empirical antimicrobial therapy	2.56	(1.35–4.87)	0.004
Early de-escalation of APBL within 48 h	0.91	(0.57–1.46)	0.71

CI: confidence intervals; APBL: antipseudomonal beta-lactams.

## Data Availability

Data are available upon request.
